# Novel anterior curved incision combined with MIPO for Pilon fracture treatment

**DOI:** 10.1186/s12891-020-03207-3

**Published:** 2020-03-18

**Authors:** Dankai Wu, Chuangang Peng, Guangkai Ren, Baoming Yuan, He Liu

**Affiliations:** 1 Department of Orthopedics, The Second Hospital of Jilin University, Changchun, 130041 China; 2 Orthopaedic Research Institute of Jilin Province, Changchun, 130041 People’s Republic of China

**Keywords:** Pilon fracture, Curved incision, MIPO, Limited open reduction

## Abstract

**Backgrounds:**

Poor prognosis was reported for complex Pilon fractures involving severe soft tissue damage. It is therefore useful to explore the evolution of different treatment strategies in an effort to reconstruct the axial alignment and articular surface, while minimizing additional damage to the surrounding soft tissues.

**Methods:**

Seventeen patients with Pilon fractures were enrolled in this retrospective study from December 2009 to October 2014. The injuries were graded according to AO Classification and the Gustilo-Anderson system. Patients were treated with minimally invasive plate osteosynthesis (MIPO) combined with curved incision on the anterior area of ankle. The ankle function and radiological outcome were assessed by the modified Mazur ankle score and Burwell-Charnley criteria, respectively. Visual analogue score (VAS) score was used to assess the degree of patient’s ankle pain, and related complications were also recorded.

**Results:**

The mean time for fracture healing was 3.6 months (range: 3–6 months). According to Mazur’s criteria, surgical treatment achieved good or excellent outcome in 15 (88.2%) cases, and the average VAS score was 1.19 ± 0.52. On the basis of Burwell-Charnley score, 12 (70.5%) patients achieved anatomic recovery, 4 (23.5%) obtained good reduction, and only 1 (5.9%) patient was diagnosed with valgus deformity. Additionally, 1 (5.9%) patient developed a superficial infection around incision, and 2 (11.8%) experienced superficial peroneal nerve damage. In addition, 2 (11.8%) patients showed radiographic evidence of existing ankle osteoarthritis at the final follow-up.

**Conclusions:**

This retrospective study is the first to assess the application of a curved incision on the anterior area of ankle with MIPO for the treatment of Pilon fractures, which achieves high functional recovery with a low complication rate. However, large randomized controlled trials comparing different approaches and fixation methods are still needed to conclusively identify the optimal treatment protocol.

## Background

Pilon fractures are comminuted distal tibial fractures involving the ankle joint surface with bone loss and cancellous bone compression; they are often combined with severe soft tissue damage [1]. This fracture type has a high rate of complications, such as infection nonunion, malunion and post-traumatic arthritis. The complex fracture pattern is an intellectual and technical challenge for treatment [2]. To obtain ideal clinical outcomes, surgery should be planned to achieve anatomic reduction and stable fixation of the articular surface, tibial alignment restoration, minimal damage to skin tissue, and preserved blood circulation [3]. Various methods for Pilon fracture treatment have been reported, such as minimally invasive plate osteosynthesis (MIPO) [4], external fixation [5], open reduction and internal fixation (ORIF) [6], and intramedullary interlocking nails [7]. However, when these classical strategies are adopted to treat high-force Pilon fracture with associated soft tissue damage, it is difficult to obtain the optimal result. In these situations, open (or limited) fracture reduction access via the anterior talocrural region may be necessary. For such cases, one or more limited incisions are needed to expose the articular key zone and perform retrograde insertion of plates for visual and stable fracture fixation (partial MIPO).

To minimize damage to the surrounding soft tissues, it is clinically meaningful to explore the evolution of different treatment strategies in an effort to reconstruct the axial alignment and articular surface originated from Pilon fractures. In this study, we developed an improved surgical approach of curved incision on the anterior area of ankle that permits visualization of the articular surface without excessive dissection of the surrounding soft tissues. The clinical effect of the curved incision in combination with MIPO for the treatment of Pilon fractures was explored for the first time in this research.

## Methods

### Inclusion criteria and exclusion criteria

1. **Inclusion criteria:** I) Adults older than 18 years and younger than 75 years, II) patients who were clinically diagnosed with Pilon fracture, III) limited exposure and internal fixation can complete the goal of anatomical reduction of the tibial articular surface, IV) no episodes of compartment syndrome, and V) Gustilo-Anderson type I/II open fractures with small open wounds mainly on the medial aspect of the distal tibia.
2. **Exclusion criteria:** I) Severe injury with Gustilo-Anderson type III open fracture, II) Pilon fractures with critical vessel and nerve injury, III) patients who cannot complete follow-up more than 6 months, or IV) severe distal tibial fracture with difficulty reconstructing the articular surface due to limited exposure.

### Study design and participants

**Fig. 1:**
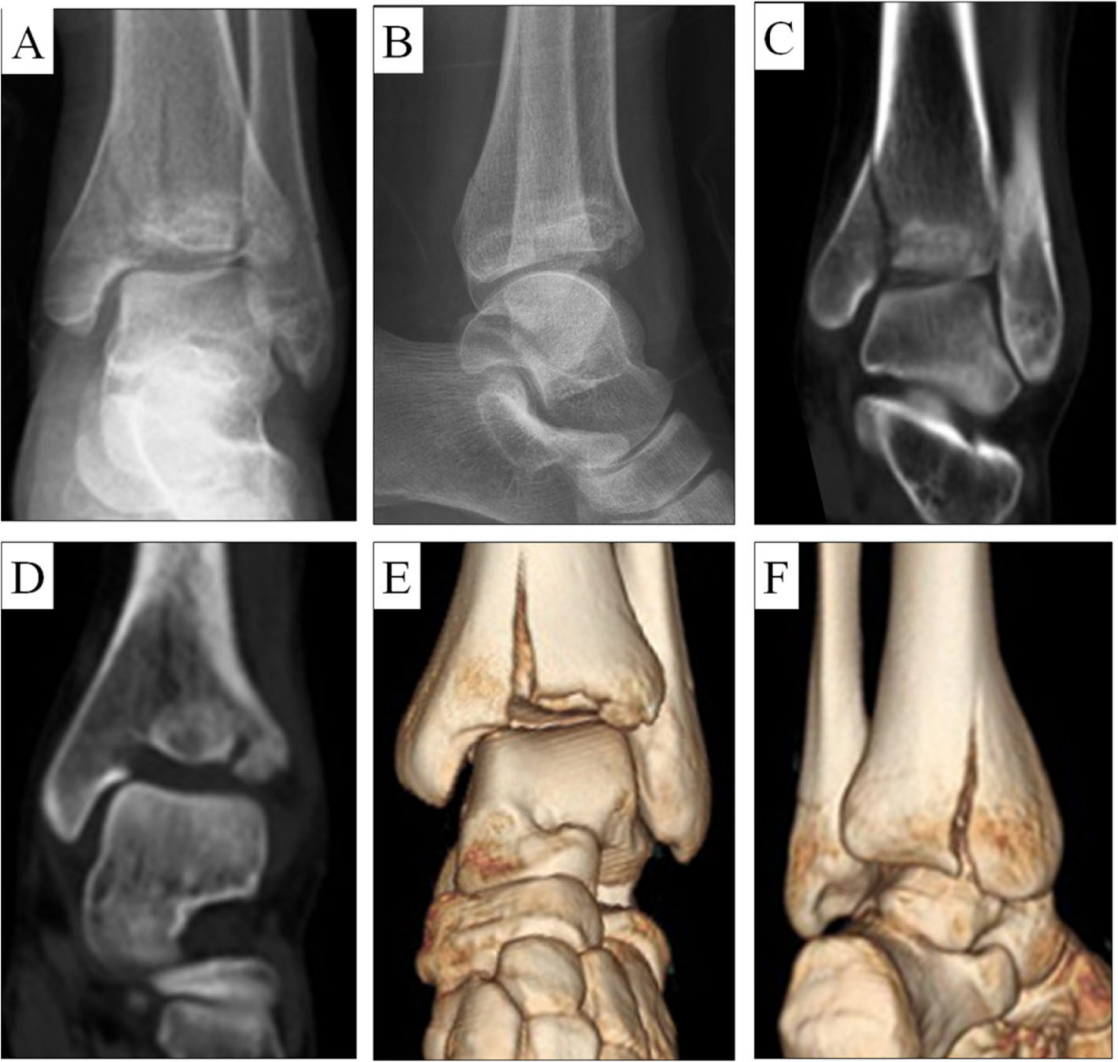
A female patient diagnosed with a 43-B2 Pilon fracture. **a**-**b** Preoperative X-rays and (**c**-**f**) three-dimensional CT showed the splitting and depression of the distal tibial articular surface. The Pilon fracture was combined with medial and lateral malleolus fractures

The study was approved by the ethics committee of the Second Hospital of Jilin University (No. 2019005). Seventeen patients diagnosed with Pilon fracture were enrolled in this study and accepted limited incision on the anterior area of ankle combined with MIPO from December 2009 to October 2014. The mean age was 37.4 years (29–62), including 11 males and 6 females. The causes of high-energy injury included traffic accidents and falling from a height. The diagnosis and classification of Pilon fractures were verified by radiographic and three-dimensional computed tomography (CT) scan images (Fig. 1). AO classification of these patients were from 43-B2 (5 cases), 43-C1 (7 cases), 43-C2 (3 cases), and 43-C3 (2 cases). There were 15 (88.2%) closed injuries, 1 (5.9%) type I injury, and 1 (5.9%) type II injury according to the Gustillo-Anderson system. In addition, 11 patients sustained multiple fractures including ipsilateral medial malleolar or distal fibular fractures. The detailed information of 17 patients and their fracture characteristics are summarized in Table 1.

**Table 1:** Patient and fracture characteristics

Index	Performance
Male/female	11/6
Age	37.4 years (29–62)
Right/left	7/10
Mechanisms of injury	
Motor vehicle crash	5 (29.4%)
Fall	12 (70.6%)
Isolated distal tibial fracture	6 (35.3%)
Multiple fractures	11 (64.7%)
Fracture type	
43-B2	5 (29.4%)
43-C1	7 (41.2%)
43-C2	3 (17.6%)
43-C3	2 (11.8%)
Involving Chaput fragment	10 (58.8%)
Closed	15 (88.2%)
Open	
Gustilo-Anderson I	1 (5.9%)
Gustilo-Anderson II	1 (5.9%)
Ankle dislocation	
Yes	7 (41.2%)
No	10 (58.8%)

### Preoperative preparation

The time interval of definitive surgery following a staged protocol is crucial in patients with Pilon fractures. Sufficient time was allowed to reduce swelling for 6 to 10 days after the initial injury. Patients with open fractures underwent debridement until no sign of infection was observed. Calcaneal traction was performed to maintain the Pilon fracture reduction and prevent further damage to the local soft tissue, only if the affected ankle was obviously swollen. Once the swelling was alleviated, the internal fixation could be performed.

### Surgical techniques

General anesthesia or combined spinal-epidural anesthesia was employed according to the patient condition, and exsanguination band was routinely applied. Eleven cases were accompanied by fibular fracture, which was handled first. Then, curved incision combined with MIPO was performed in three steps (i.e.*,* curved incision on the anterior area of ankle, fracture reduction and bone graft, and internal fixation), which are described in detail as follows.

**Fig. 2:**
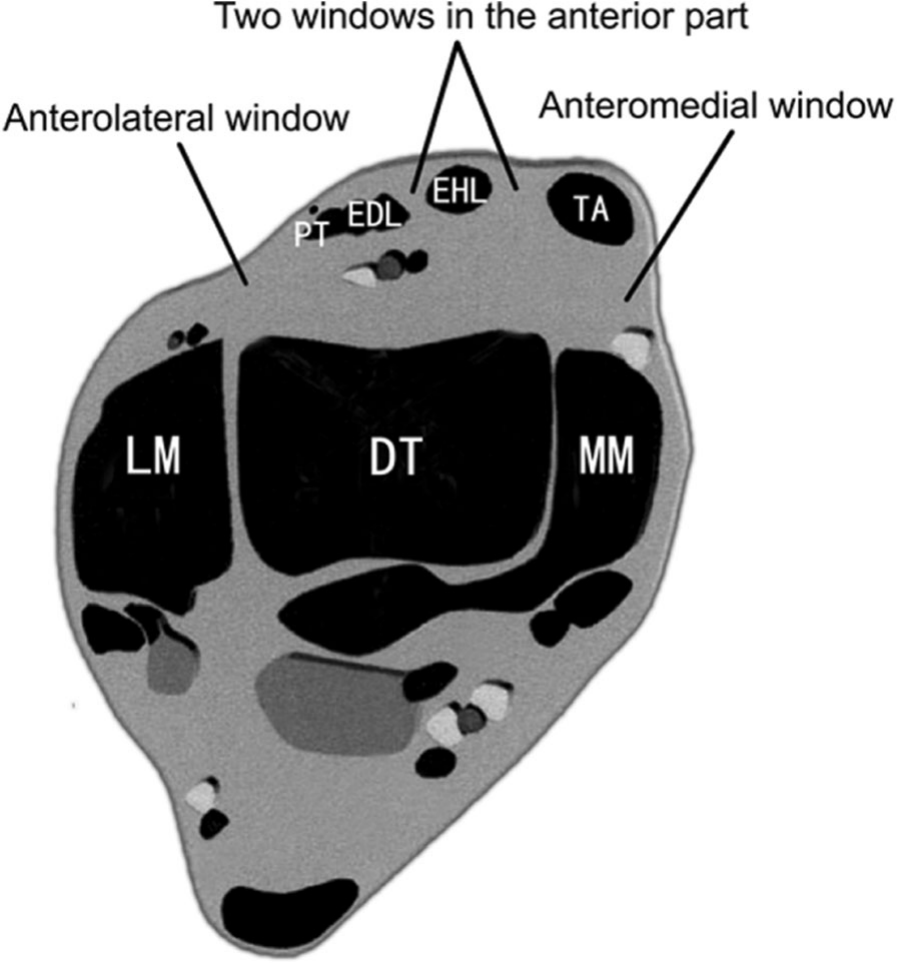
Transverse image of the ankle showing the relationships among the tendons and four small soft-tissue windows. PT, peroneus tertius; EDL, extensor digitorum longus; EHL, extensor hallucis longus; TA, tibialis anterior; LM, lateral malleolus; DT, distal tibia; MM, medial malleolus

**Fig. 3:**
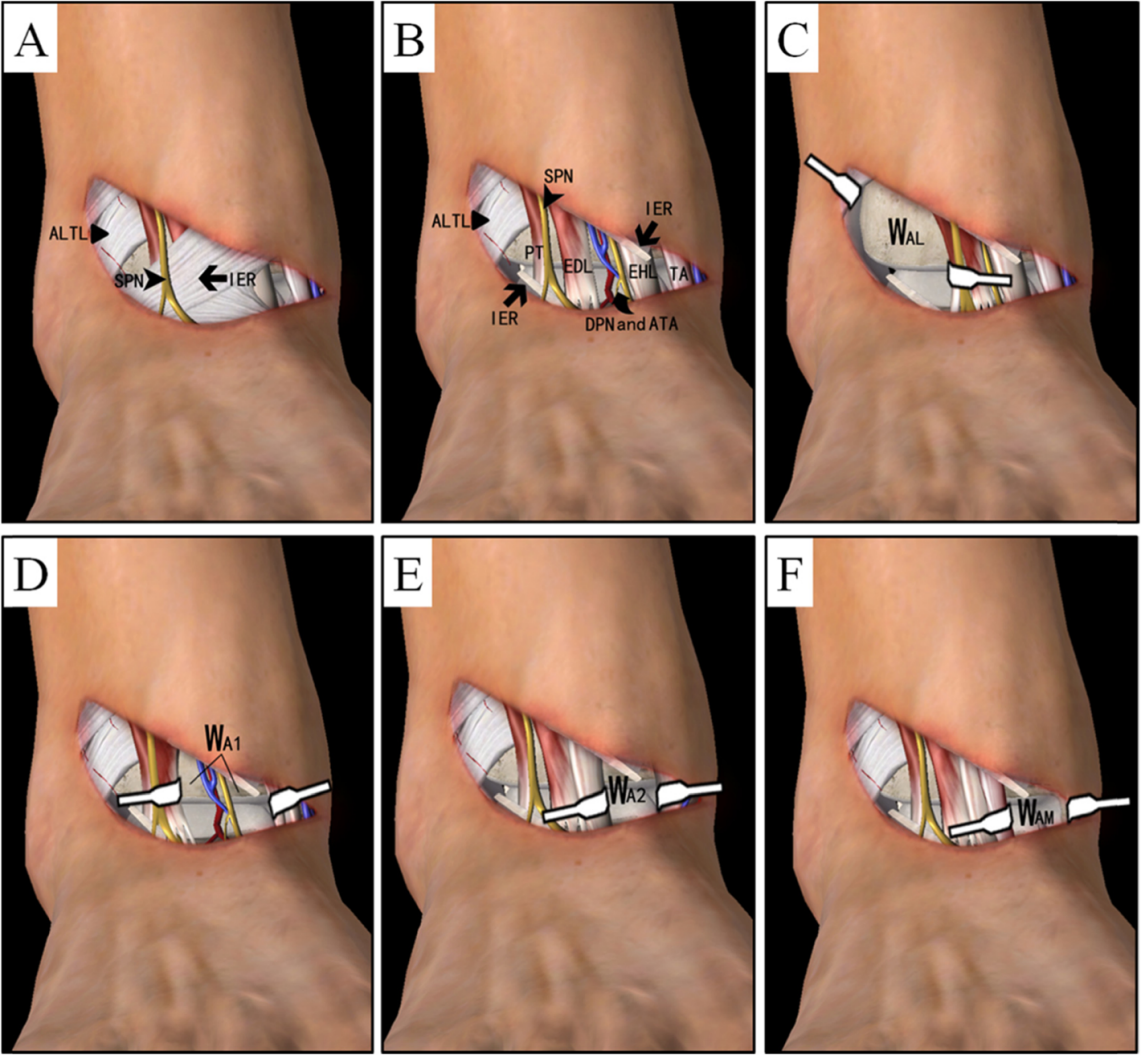
**a** A curved incision was made through the intermuscular interval. **b** The medial extensor support band was horizontally cut, and the lateral band was partly cut to maintain its overall integrity. **c** One anterolateral window, namely W_AL_, **d**, **e** two anterior windows, namely W_A1_ and W_A2_, **f** one anteromedial window, namely W_AM_. ALTL, anterior lower tibiofibular ligament; SPN, superficial peroneal nerve; IER, inferior extensor retinaculum; PT, peroneus tertius; EDL, extensor digitorum longus; EHL, extensor hallucis longus; TA, tibialis anterior; DPN and ATA, deep peroneal nerve (DPN) and anterior tibial artery (ATA)

- **Step 1.** Curved incision on the anterior area of ankle: A curved incision was applied starting from the straight tip of the medial malleolus and extending 6–8 cm outwards to the tibiofibular syndesmosis. The flexor retinaculum was identified and incised horizontally. Attention was paid to avoid superficial peroneal nerve injury at the incision site. In deep tissue, the deep peroneal nerve (DPN) and anterior tibial artery (ATA) pass longitudinally along the incision, which were carefully identified and protected. Anterior tendons were identified from medial to lateral (e.g.*,* the tibialis anterior [TA], extensor hallucis longus [EHL], extensor digitorum longus [EDL], and peroneus tertius [PT]) (Fig. 2). Careful blunt dissection over the anterior edge of the distal tibia was performed to expose the articular capsule. Four small soft-tissue windows could be created using the above intermuscular interval to access the entire articular surface, including one anteromedial window, two anterior windows, and one anterolateral window (Fig. 3).
- **Step 2.** Fracture reduction and bone graft: Alignment correction and fracture reduction are the most crucial procedures before percutaneous plating for metaphyseal injuries. The articular surface could be temporarily fixed by K-wires to restore articular surface using the talar articular surface as the template. Additional structural support with bone transplantation could be performed under direct vision through the four windows described in Step 1.
- **Step 3.** Internal fixation: In all cases, the anterolateral distal tibia locking compression plate (LCP) was chosen for fracture fixation. The plate was retrogradely inserted through the anterolateral window between the peroneus tertius and lateral malleolus and placed under the anterior compartment. The distal screws were inserted under direct vision in a raft pattern to provide stable support for the elevated articular fragments. The proximal 3–4 screws were inserted percutaneously, with a similar sized LCP to reference the hole positions (mirror plate technique). Any medial involvement such as a medial malleolus fracture could be addressed with a small anteromedial incision and lag screws. In one case, the medial malleolar fracture had to be fixed using a reshaping reconstruction plate to allow stable anchoring of short unicortical locking screws distally.

### Postoperative treatment and rehabilitation program

To achieve early functional restoration, passive and active motion of ankle joint must be performed as soon as possible. The patients started partial weight bearing (10–15 kg) as tolerated in accordance with the patient’s general condition. Full weight bearing was recommended after X-ray showing the fracture was consolidated. Implant removal can be performed 1–2 years after the remodeling process has finished. Younger patients might require removal, but elderly patients can better tolerate implants, so routine metal removal may not be recommended.

### Postoperative assessments

All patients underwent routine evaluations including clinical and radiographic examinations, and goniometric range of motion evaluations at 1, 3, 6, and 12 months postoperative, then once a year regularly. Fracture healing time and complications including delayed union, nonunion, and malunion were recorded. During the follow-up period, each imaging examination was measured and analyzed by three doctors. The postoperative standards of fracture reduction were anatomic, good, and poor according to Burwell and Charnley [8]. Ankle function was measured with the modified Mazur ankle score. The maximum score was 100 points, and > 87 was considered to be a good to excellent result [9]. Visual analogue score (VAS) was used to assess the degree of patient’s ankle pain, and related complications were also recorded.

## Results

**Fig. 4:**
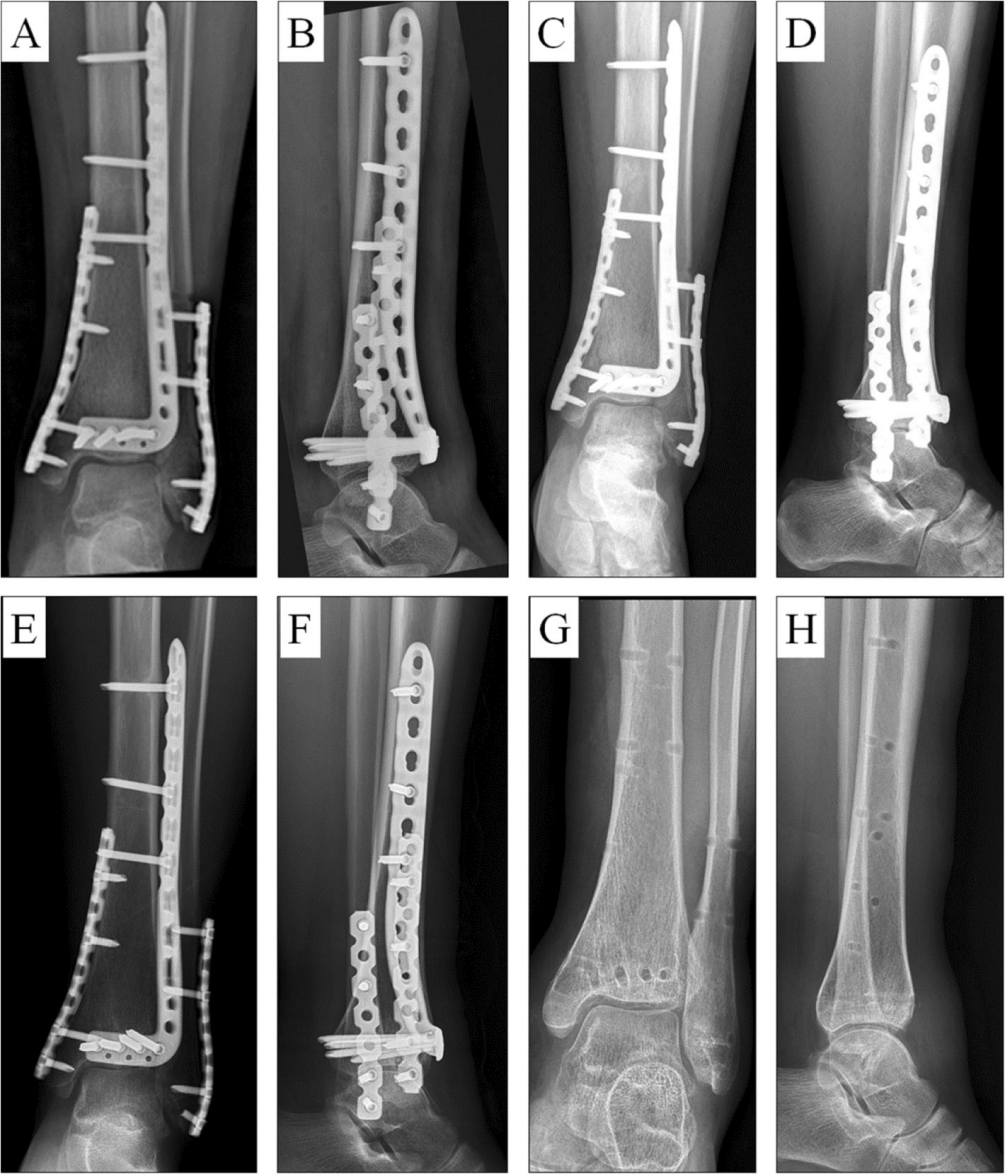
Postoperative AP and lateral X-ray examinations of the patient at **a**, **b** 1 month, **c**, **d** 3 months, and **e**, **f** 6 months postoperatively, as well as **g**, **h** after implant removal

**Fig. 5:**
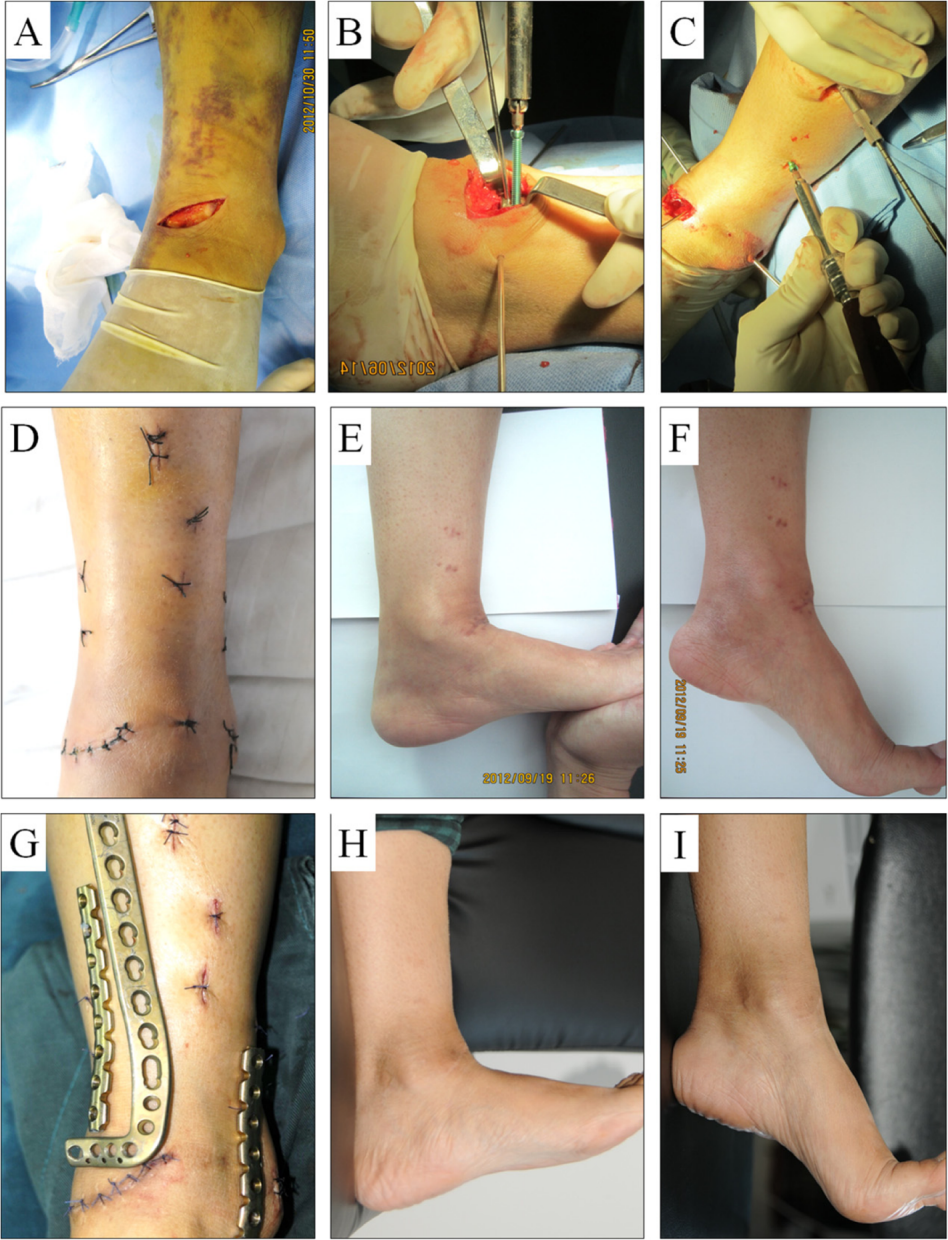
The patient was treated with the curved incision on the anterior area of ankle using two reconstruction LCPs with MIPO. **a**-**d** Intra-operative images, including curved incision, screw implantation, and postoperative incision. The patient achieved good functional outcomes at **e**, **f** 3 months and **h**, **i** 6 months after surgery, and **g** the implants were successfully removed

All 17 patients completed long-term follow up for an average 46.7 months (range: 24–72 months) as described in Table 2. The average duration between trauma and surgery was 7.6 days (range: 6–10 days). Bone union was achieved in all cases, and the mean time for radiological union was 3.6 months (range: 3–6 months) (Fig. 4). According to Burwell and Charnley radiological studies, 12 (70.5%) patients had an anatomic reduction, 4 (23.6%) had a good reduction, and 1 (5.9%) had a poor reduction. No patient experienced loss of reduction or fixation, and the mean articular step off was 1.0 mm (range: 0–3 mm). Implant removal was performed in 14 patients (82.4%), and the final follow-up showed the ankle’s range of motion was in good condition (Fig. 5). The Mazur ankle score standard was used to evaluate ankle function, which was excellent or good in 15 (88.2%) cases.

**Table 2:** Postoperative complications and outcomes

Index	Performance
Average follow-up period, months	46.7 (24–72)
Average duration between trauma and surgery, days	7.6 (6–10)
Radiographic healing time, months	3.6 (3–6)
Radiographic evaluation	
Anatomic reduction	12 (70.5%)
Good reduction	4 (23.6%)
Poor reduction	1 (5.9%)
Complications	
Articular step off	1.0 mm (range: 0–3 mm)
Superficial infection	1 (5.8%)
Superficial peroneal nerve damage	2 (11.8%)
Valgus deformity	2 (7° and 8°)
Varus deformity	0 (0%)
Arthritis	2 (11.8%)
Screw loosening and implant failure	0 (0%)
Good and excellent rate of ankle joint function (Mazur ankle score)	15 (88.2%)
VAS score	1.19 ± 0.52
Implant removal	14 (82.4%)

**Fig. 6:**
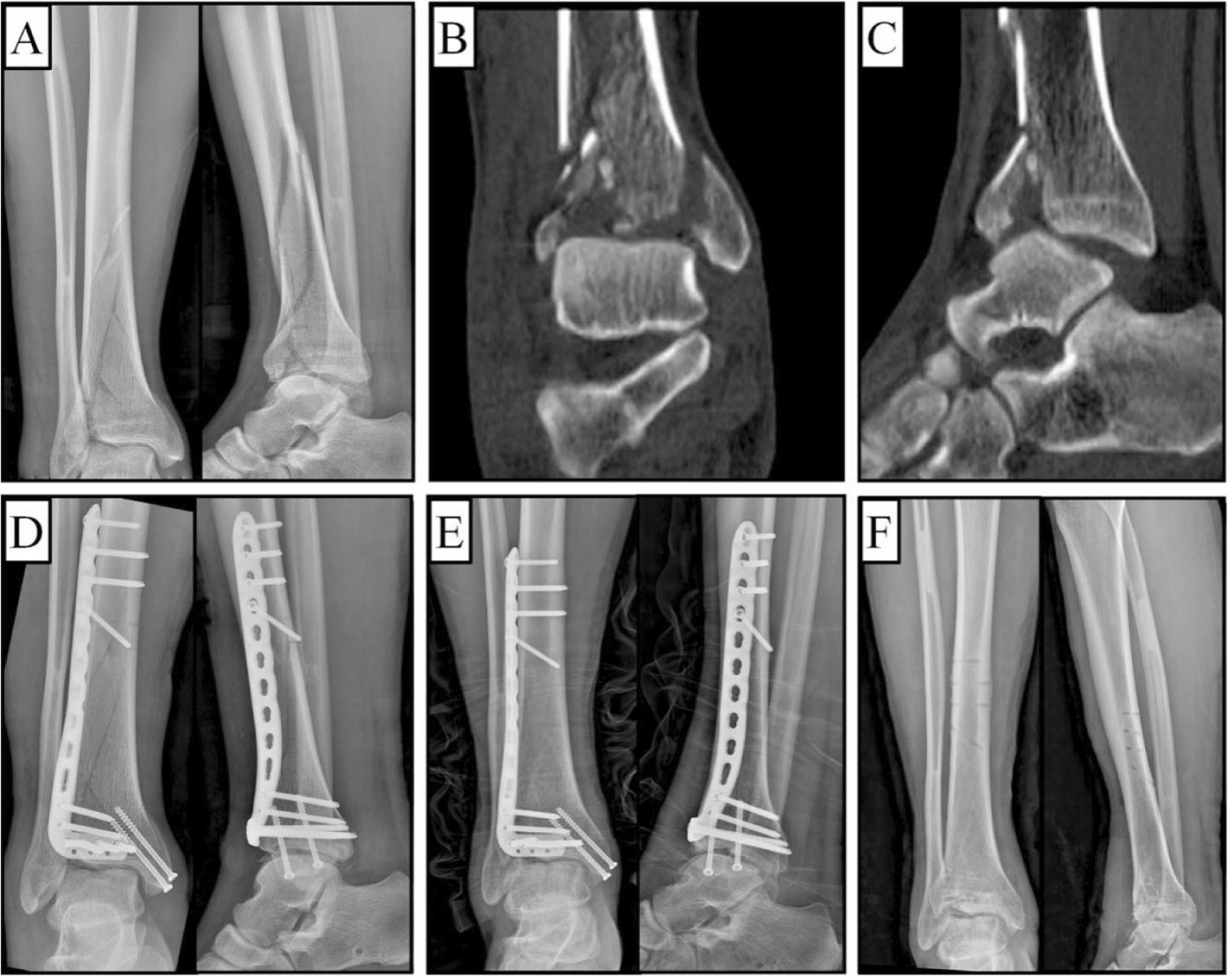
A male patient diagnosed with a 43-C1.1 Pilon fracture. **a** Preoperative X-rays, **b**, **c** coronal and sagittal CT showed spiral fracture of distal tibia and comminuted fracture of anterior distal tibia, and longitudinal fracture of the medial malleolus, as well as slight compression of the talar articular. Postoperative AP and lateral X-ray examinations of the patient at **d** 1 day, **e** 1 year postoperatively, and **f** after implant removal indicating slight arthritis

No patients experienced limb discrepancy, ankle instability, ankle joint stiffness, or dislocation. The average VAS score of involved patients was 1.19 ± 0.52. We encountered superficial infection in 1 (5.8%) case that resolved with appropriate antibiotic treatment. Subtle superficial peroneal nerve damage was observed in 2 (11.8%) patients, but they recovered following neurotrophic treatment. Clinical examination revealed misalignment exceeding 5° in the frontal plane in 2 (11.8%) patients (valgus 7° and 8°). Radiological studies showed no evidence of arthritis in 15 (88.2%) patients and slight arthritis in 2 (11.8%). A 37-year-old male patient diagnosed with complex 43-C1.1 Pilon fracture were present in Fig. 6a-c, and immedicate postoperative X-ray examinations (D) showed that distal tibial and medial malleolus fractures achieved anatomical reduction. However, X-ray examinations of one-year follow-up (E) and after implant removal (F) indicated slight arthritis.

## Discussion

In this study, an innovative curved incision on the anterior area of ankle was proposed, which was characterized with minimal soft tissue damage and flexible operation windows. Combining with MIPO technology, this novel technique achieved high functional recovery with a low complication rate for the treatment of Pilon fractures. The goals of reduction and internal fixation of Pilon fractures include reconstruction of the articular surface, restoration of mechanical alignment, and stable fixation to allow early joint motion [10]. The most frequent operative methods include ORIF, intramedullary fixation, plate fixation, and external skeletal fixation. However, poor prognosis was reported for complex Pilon fractures involving severe soft tissue damage and a large amount of bone compression [11]. It is therefore useful to explore the evolution of different treatment strategies in an effort to reconstruct the axial alignment and articular surface while minimizing additional damage to the surrounding soft tissues [12].

Minimally invasive techniques have been developed to minimize soft tissue damage. However, they are not adequate to visualize the joint surface and achieve an anatomic reduction [13]. In this study, we applied a novel curved incision of the anterior ankle. This approach allows the surgeon to visualize and anatomically reduce the articular surface, which can also minimize soft tissue damage as much as possible. After reducing the articular surface, it is bridged to the diaphysis using a minimally invasive technique. The appropriate length plate completed the reduction and internal fixation of the metaphyseal component. Restoration of length, axial alignment, and rotation enabled absolute or relative stability. It is an optimal combination of minimally invasive and limited open techniques for articular surface visualization, and we observed a significantly lower rate of postoperative complications compared with other studies [14, 15].

To apply the curved incision in the anterior region, it is necessary to fully understand the anatomy of the anterior tibiofibular region [16]. The surgeon should pay attention to protecting the anterior tibiofibular arteries and the deep peroneal nerve between the anterior tibial tendon and long extensor tendon. During surgery, the soft tissue window should be fully utilized to reconstruct the distal tibial articular surface.

The advantages of this approach are summarized as follows: I) The incision was cut along the skin texture with good blood supply, so skin necrosis and non-healing rarely occurred. II) The medial extensor support band was horizontally cut, and the lateral band was partly cut to maintain its overall integrity. Even if no intervention was performed, there was no risk of tendon jump. III) The soft tissue and blood supply were well protected to enable fast healing of fractures and soft tissue. IV) MIPO technology can maximize the blood supply to the fracture, promote fracture healing, reduce the risk of infection and re-fracture, and maintain the stability of the fracture to reduce the need for bone grafting.

There are four precautions for this technology: I) It is easier to achieve fracture reduction using the cortical bone margin for compressive Pilon fracture. II) The articular cartilage and part of the metaphyseal bone may obviously displace to the proximal segment, if the central part of the distal tibia was destroyed. At this time, the fracture block should be pulled down and restored, and the talus articular surface should be used as a template to reconstruct the joint relationship. Application of a small bone hook can restore the vertical displacement of the joint bone fragment and recover the flatness of the articular surface. III) In the actual operation, the corresponding tendons must be repeatedly pulled to expose the articular surface and the displaced bone in the inner and outer sides, which may damage the anterior iliac vessels and deep peroneal nerves. IV) Appropriate early exercise is helpful to the joint stress stimulus; it gradually transforms the granulation tissue at the bone defect into bone tissue, fibrocartilage, and even hyaline cartilage, which is beneficial to the healing and grinding of the articular surface, reducing the risk of traumatic arthritis.

## Conclusions

To the best of our knowledge, this retrospective study is the first to assess the application of a curved incision on the anterior area of ankle with MIPO. Based on our observations, this method achieved high functional recovery with a low complication rate for the treatment of Pilon fractures. Our results should be confirmed in randomized controlled trials in larger patient sample comparing different approaches and fixation methods to conclusively identify the optimal treatment protocol.

## Data Availability

Data from the Department of Orthopedics, the Second Hospital of Jilin University. The datasets used and/or analyzed during the current study are available from the corresponding author on reasonable request.

## Abbreviations

ALTL: Anterior lower tibiofibular ligament
ATA: Anterior tibial artery
CT: Computed tomography
DPN: Deep peroneal nerve
EDL: Extensor digitorum longus
EHL: Extensor hallucis longus
IER: Inferior extensor retinaculum
LCP: Locking compression plate
MIPO: Minimally invasive plate osteosynthesis
ORIF: Open reduction and internal fixation
PT: Peroneus tertius
SPN: Superficial peroneal nerve
TA: Tibialis anterior
W_AL_: Anterolateral window
W_A1_ and W_A2_: Anterior windows
W_AM_: Anteromedial window
VAS: Visual analogue score
